# *head-bent* resistant Hsc70 variants show reduced Hsp40 affinity and altered protein folding activity

**DOI:** 10.1038/s41598-019-48109-0

**Published:** 2019-08-16

**Authors:** Katharina Papsdorf, Siyuan Sima, Lukas Schmauder, Sebastian Peter, Lisa Renner, Patrica Hoffelner, Klaus Richter

**Affiliations:** 1 Center for integrated protein research at the Department of Chemie, Technische Universität München, Lichtenbergstr. 4, 85748 Garching, Germany; 2 Present Address: Stanford University School of Medicine, Department of Genetics, 300 Pasteur Drive, Stanford, CA 94305 USA

**Keywords:** Chaperones, Oncogene proteins

## Abstract

The molecular chaperone Hsc70 performs essential tasks by folding proteins. Hsc70 is driven by the hydrolysis of ATP and tuned by the association with various co-chaperones. One such cofactor is the nematode nucleotide exchange factor UNC-23, whose mutation disrupts muscle attachment and induces a severe *head-bent* phenotype in *C.elegans*. Interestingly, four mutations in Hsc70 can suppress this phenotype, but the molecular mechanism underlying this suppression is unknown. Here we characterize these four suppressor variants, Hsc70 D233N, S321F, A379V and D384N. *In vitro* only Hsc70 S321F shows reduced stability and altered nucleotide interaction, but all mutations affect the ATPase stimulation. In particular, Hsc70 D233N and Hsc70 A379V show strongly reduced interactions with DNJ-12 and DNJ-13. Nucleotide exchange factor binding instead is barely influenced in Hsc70 D233N, A379V and D384N and their chaperone activity is preserved. Molecular dynamics simulations suggest that effects in Hsc70 S321F and Hsc70 A379V originate from steric clashes in the vicinity of the mutation site, while D233N disrupts a salt bridge that contributes to Hsc70’s nucleotide-induced conformational changes. In summary, the analyzed mutants show altered ATPase and refolding activity caused by changes in Hsp40 binding.

## Introduction

The highly conserved molecular chaperone Hsc70 assists client protein folding in bacteria, fungi and higher eukaryotes. It promotes *de novo* folding of a diverse client set under physiological and stress conditions^1,2^ ensuring general protein homeostasis^3,4^. Hsc70 is an ATPase with an N-terminal nucleotide binding domain (NBD) and a C-terminal substrate binding domain (SBD), which is covered by a flexible substrate lid. Hsc70 interacts with various cofactors that modulate client interaction and tune ATP hydrolysis. For example, Hsp40 proteins present the client protein to Hsc70 and accelerate ATP hydrolysis by interaction with the ATP bound Hsc70 conformation^5^. The co-chaperone group of Nucleotide Exchange Factors (NEF) instead compete with Hsp40 proteins to access Hsc70 and subsequently initiate the release of nucleotide and client from the complex^6^. In *C. elegans* Hsc70’s ATP turnover is influenced by the Hsp40-like J-domain cofactors DNJ-13 and DNJ-12 and the NEFs UNC-23 and BAG-1^7–9^. The combination of Hsp40s and NEFs synergistically dramatically increases Hsc70’s ATP turnover and client folding in all organisms studied to date^8,10–12^.

The *hsp-1* gene encodes the sole functional Hsc70-like protein in the nematode’s genome. This stands in contrast to many other organisms, such as yeast, where several functional homologs exist^13,14^. This offers the unique possibility to study the involvement of this essential chaperone in physiological processes. Knockdown experiments and homozygous deletion of *hsp-1* in *C. elegans* show that nematode Hsc70 is critically required already during early larval development^15,16^. Interestingly, overexpression of a fluorescently tagged HSP-1 leads to locomotion defects that are related to defects in the muscular attachment sites such as the *head-bent* phenotype^7^. This locomotion defect is phenocopied by mutations of the Hsc70 co-chaperone *unc-23*^17,18^. Here, degeneration of the head muscle cells leads to the failure of forward movements and conditions that resemble variations of muscular dystrophy^17,19^. This implies that Hsc70 complexes are influencing the attachment of muscle cells possibly by providing protection against shear stress^17,19^. Interestingly, mutation of multiple loci of *unc-23* induce this strong detrimental phenotype such as *unc-23(e25)*, which encodes the point mutation UNC-23 E297K, and *unc-23(ok1408)* which is characterized by a large deletion within *unc-23*. Recently four viable mutations in the ATPase domain of *C. elegans* Hsc70 were uncovered, which confer resistance to the *unc-23* mutation induced *head-bent* phenotype^17,20^. Two such mutations, Hsc70 D233N and Hsc70 A379V, were identified in a mutagenesis screen with the *unc-23(e25)*-allele encoding UNC-23 E297K^17^. In an independent study, two other mutations, Hsc70 S321F and Hsc70 D384N, were identified to confer resistance to the deletion allele *unc-23(ok1408)*^20^. However, the underlying molecular mechanism of how the point mutations in the essential chaperone Hsc70 can rescue the drastic locomotion phenotype is not understood.

**Figure 1:**
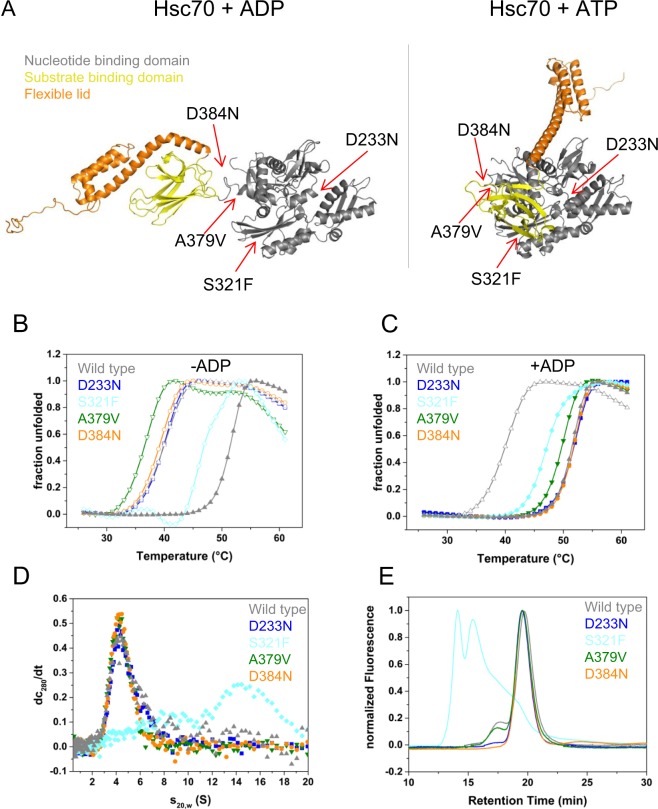
Structure and Stability of the Hsc70 mutants. (**A**) Closed Hsc70 structure with the four mutation sites indicated as D233N, S321F, A379V and D384N. ADP bound DnaK (PDB: 2KHO) with closed substrate binding domain served as template for the model. The NBD is shown in grey, the SBD in yellow, the lid domain in orange. Mayor rearrangements of the domains towards each other in ATP bound BIP (PDB: 5E84) with the same color code as before. (**B**) Structure and stability of Hsc70 mutants in the absence of nucleotide. Tertiary structure determination was performed using the thermal shift assay (TSA). Samples contained Hsc70 with (gray ▲) and without ADP (gray △) and Hsc70 D233N (blue □), Hsc70 S321F (cyan ◊), Hsc70 A379V (olive ▽) and Hsc70 D384N (orange ○) in standard buffer. (**C**) Structure and stability of the Hsc70 mutants in the presence of ADP. Samples contained Hsc70 with and without ADP and the variants Hsc70 D233N (blue ▪), Hsc70 S321F (cyan ◆), Hsc70 A379V (olive ▼) and Hsc70 D384N (green ●) in standard buffer with 1 mM ADP. (**D**) Analytical ultracentrifugation reporting on the quaternary structure of the variants. Sedimentation velocity experiments were performed and dc/dt plots were generated as outlined in the Material and Methods section. Identical concentrations of Hsc70 (gray), Hsc70 D233N (blue), Hsc70 S321F (cyan), Hsc70 A379V (olive) and Hsc70 D384N (orange) were used. (**E**) SEC-HPLC was performed in standard buffer as described in the Material and Methods section. Line colors are as described in (**D**).

While all these Hsc70 mutants are localized in the NBD (see Fig. 1A) it is not understood which changes in the cofactor interaction pattern they induce and if potentially they result in changes in Hsc70’s activity. Here we characterize the Hsc70 mutations to understand, how phenotypic suppression could originate in this system. We find mechanistic changes during the ATPase cycle of these Hsc70 suppressor mutations likely driven by reduced cofactor binding. This largely correlates with the genetic interaction between Hsc70 and its cofactors UNC-23 and DNJ-13 *in vivo*.

## Materials and Methods

### Nematode handling

Nematodes were handled according to standard procedures^21^. *unc-23(e25)*, *unc-23(e324)* and *unc-23(ok1408)* strains (CB25, CB324, RB1301) were obtained from the CGC and were grown on NGM plates seeded with OP50 bacteria at 20 °C. For RNAi experiments HTT15(DE3) bacteria were freshly transformed with the dsRNA-encoding plasmids and then used as sole feeding source on RNAi plates. In these experiments synchronized L1 nematodes were transferred to plates containing NGM supplemented with 100 µg/ml ampicillin, 6 µg/ml tetracycline and 1 mM IPTG. Genes targeted by this approach were *dnj-13*, *dnj-12* and *dnj-19*^15,22^ and the results were compared to empty L4440 vector control plasmid. To measure the nematode’s motility thrashing assays were performed by transferring nematodes at adult day 1 to a droplet of M9. The thrashing movements per minute at 20 °C were recorded for 20 animals.

### Protein expression

Wild type Hsc70 was expressed from a pET28b-Hsc70 expression plasmid and purified as described^8^. All mutations in nematode Hsc70 were generated by QuickChange PCR using the plasmid pET28b-Hsc70 and primers designed with the help of NEBaseChanger (https://nebasechanger.neb.com/). Resulting plasmids were sequenced to confirm the mutation (GATC-Biotech, Konstanz, Germany). Protein expression was performed in 5 L flasks at 20 °C for 16 h and protein purification was performed as previously described for Hsc70^7,8^. In brief, His-tagged proteins were purified by affinity purification via a Ni-NTA column, followed by ion-exchange and size exclusion chromatography. Hsc70 variants were stored in 40 mM HEPES/KOH pH 7.5, 50 mM KCl at −80 °C. Protein identity and purity were confirmed by mass spectrometry and SDS-PAGE. Hsc70 co-chaperones were purified as reported^8^. Purified co-chaperones were stored in 40 mM HEPES/KOH, pH 7.5, 150 mM KCl, 1 mM DTT, 1 mM EDTA. Ydj1 was purified as a His-SUMO-tagged protein. Here, after Ni-NTA affinity purification, the His-SUMO tag was cleaved by SUMO-protease and removed via Ni-NTA affinity purification followed by size exclusion chromatography. Luciferase was expressed in autoinduction medium^23^ and purified using DEAE-Sephadex and size exclusion chromatography. Luciferase activity assays were employed to select the luciferase-containing fractions. Purified luciferase stocks were stored in PBS at −80 °C.

### ATPase assay

The ATPase activity of Hsc70 was determined using an NADH coupled regenerative assay with 3 µM Hsc70 as described^24^. Measurements were performed at 25 °C in 120 µL cuvettes in 40 mM HEPES/KOH pH 7.5, 150 mM KCl, 5 mM MgCl_2_. Baseline activity was determined and the assay started by addition of 2 mM ATP. The ATPase activity was determined in OriginPro 8.6 (OriginLabs, Northampton, USA) with the following equation, in which $\varepsilon (NA{D}^{+})-\varepsilon (NADH)=-\,6200/({\rm{M}}\ast {\rm{cm}})$.

$$Activity=\frac{\frac{{\rm{\Delta }}{A}_{340}}{{\rm{\Delta }}t}}{(\varepsilon (NA{D}^{+})-\varepsilon (NADH))\cdot c\,(ATPase)}$$

To determine the stimulation of the ATPase activity by co-chaperones, DNJ-13, DNJ-12, BAG-1 and Δ258-UNC-23 were added before the addition of ATP at the indicated concentrations. The extent of stimulation was calculated by subtracting the not stimulated ATPase rates of Hsc70s from the stimulated rates. This yields a value, which is free from potential non-Hsc70 related background, even if the background originates from the Hsc70-preparations. In cases, where the co-chaperone preparations showed ATPase activity, this additional background activity was also subtracted. Standard deviations were combined as required for subtraction of error-containing data. Each assay was measured three times and the average stimulation with standard deviation is plotted. Comparison of two sample t-test was performed to analyze statistical significance.

### Analytical ultracentrifugation

Sedimentation velocity experiments were carried out in a Beckman XL-A analytical ultracentrifuge equipped with an AVIV AU-FDS fluorescence detection unit (AVIV Biomedical, New Jersey) as described^8^. To this end *BAG-1 was generated by fluorescently labeling BAG-1 at its cysteine residue with ATTO 488 C_5_-maleimide (ATTO-TEC GmbH, Siegen, Germany). DNJ-13 was expressed with an additional cysteine in the His-tag linker and selectively labeled with ATTO 488 C_5_-maleimide at this position. Δ258-UNC-23 was labeled with Alexa Fluor 488 C_5_-maleimide (Thermo Fisher Scientific) and DNJ-12 was labeled with ATTO-488-NHS (*DNJ-12) for 2 hours at room temperature. Proteins were subsequently dialyzed to remove the free label. For binding analyses 300 nM of *BAG-1 or *DNJ-12 or *600 nm of DNJ-13 were sedimented at 42,000 rpm, 20 °C in the absence or presence of binding partners. Sedimentation velocity experiments were performed in 40 mM HEPES/KOH pH 7.5, 150 mM KCl, 1 mM DTT and 5 mM MgCl_2_. 1 mM of nucleotides were added as indicated. dc/dt distributions were visualized with the program Sedview^25^ and the customized script diffUZ was used for flexible scan range selection, for normalization of the data and for generation of the plots as described previously^7,8^. Fits to Gaussian functions were made in OriginPro 8.6 (OriginLabs, Northampton, USA). Each assay was measured in triplicates and a representative graph is plotted.

Characterization of the oligomerization status of Hsc70 variants was performed with the UV/VIS optical system (Beckman Coulter). Protein samples containing 0.5 mg/mL protein were used to determine the sedimentation coefficients of the Hsc70 variants. Data analysis was based on the generation of dc/dt plots with customized scripts. UltraScan II^26^ was used to get information on the heterogeneity of the sample and to derive diffusion coefficients and molecular weights for the species.

### Size-Exclusion HPLC

Size-Exclusion HPLC was performed on a HPLC system consisting of a Jasco PU-980 pump coupled to a Jasco UV-975 detector. A Superdex 200HR (GE Amersham) column was used to perform the separation in a buffer of 40 mM HEPES, pH 7.5, 150 mM KCl. 10 µg of Hsc70 variants were injected and the elution was recorded. Data was exported and plotted with Origin 8.6 (OriginLabs, Northampton, USA).

### Thermal shift analysis

The melting temperature of the Hsc70 variants was determined in thermal shift assays (TSA). To this end, a 1:1000 fold dilution of SYPRO Orange (Thermo Fisher) was added to 0.2 mg/ml sample protein in 20 µL 40 mM HEPES/KOH pH 7.5, 150 mM KCl, 2 mM MgCl_2_, 1 mM DTT. Fluorescence was measured in a fluorescence plate reader (Agilent Stratagene Mx3005P, excitation filter: 470 nm, emission filter: 570 nm) at an 1 °C/min heating rate. Nucleotides were added as indicated. Data analysis was performed with OriginPro 8.6 (OriginLabs, Northampton, USA). Each assay was measured in triplicates and a representative graph is plotted.

### Luciferase refolding

The refolding capacity of the Hsc70 variants was investigated in a luciferase refolding assay^12,27^. To this end luciferase was chemically denatured in 25 mM HEPES/KOH pH 7.5, 50 mM KCl, 15 mM MgCl_2_, 10 mM DTE, 1 mM ATP, 50 µg/mL BSA, 5 M urea for 45 minutes. Refolding was initiated by 125-fold dilution into 25 mM HEPES/KOH pH 7.5, 50 mM KCl, 15 mM MgCl_2_, 2 mM DTE, 240 µM CoA, 1 mM ATP, 50 µg/ml BSA, 100 µM luciferin, 10 mM PEP, 50 µg/ml pyruvate kinase. The regained luciferase activity was measured continuously by recording luminescence in 96-well plates (Greiner Microlon, Solingen, Germany) in a PHERAstar^Plus^ (BMG LabTech) for 4 hours at 25 °C. If not indicated otherwise 3.2 µM of wild type and mutant Hsc70 were used with 0.8 µM of the Hsp40-like cofactors Ydj1, Hdj1, DNJ-12 or DNJ-13. Due to much lower activity, the DNJ-13 and DNJ-12 containing assays had to be corrected by subtracting the background signal of the respective Hsc70 variant, which were not identical. This effect is neglectable for the highly active Ydj1 and Hdj1 proteins, but becomes relevant, when comparing the Hsc70 variants with DNJ-13 and DNJ-12.

### Influence of mutations on Hsc70 protein stability

Homology modeling was applied to generate the structures of docked and undocked nematode Hsc70 based on crystal structures of the respective Hsc70 conformations from other organisms. We used the ATP-bound state of human BIP (PDB: 5E84)^28^ and yeast Sse1 (PDB: 2QXL)^29^ and Sse1 bound to bovine Hsc70 (PDB: 3C7N) as “docked” conformations and we used *E.coli* DnaK (PDB: 2KHO)^30^ and bovine Hsc70 bound to yeast Sse1 (PDB: 3C7N) as “undocked” conformation^31^. For homology modeling a sequence alignment was generated with ClustalOmega^32^ based on Hsc70, Hsp70, BIP and Hsp110 proteins from *E. coli*, *S. cerevisiae*, *C. elegans, B. tauris* and *H. sapiens*. This alignment was used in Chimera Modeller^33^ to calculate the structural coordinates of nematode Hsc70 in the docked (ATP bound) and undocked (ADP bound) conformation. Chimera Modeller generated an ensemble of five independent structures for each starting structure, yielding 15 structures for the docked state and 10 structures for the undocked state altogether. In the undocked models, the distance between the NBD and SBD differed, which is caused by the flexibility of the ~10 amino acid linker connecting the two domains. The ATPase domains of all generated structures were visually compared in PyMol^34^ to assess whether they had converged similarly. Indeed within the docked ensemble the root mean square deviation (RMSD) of the ATPase domains was found to be between 0.5 Å (modeled homologs of the same PDB structure) and 1.6 Å (models with different PDB starting structure), while significant differences were visible towards the undocked models (RMSD >4 Å). As all investigated mutations reside in the NBD of Hsc70, only the modeled ATPase domains up to position 389 were used for further computational analysis.

To compare the influence of the mutations, single amino-acid mutations were introduced with Chimera^33^ and with the FoldX BuildModel tool^35^. This way each mutation yielded an energetic penalty value relative to the wild type protein. This analysis was performed for the modeled structures generated. Thus, each mutation was quantified 25 times, with 5 calculations derived from the same PDB starting structure. These values were used to calculate the standard deviations depicted in the respective figures. The BuildModel tool displayed contributions to stability from different biophysical parameters, including electrostatic and hydrophobic contributions. The electrostatic values were used to plot the contribution of charged interactions to overall stability.

### Simulation of the influence of mutations on the Hsc70 dynamics

Molecular dynamics simulations were performed to visualize the influence of the mutations onto the structure and stability of the respective Hsc70 variant. To this end the isolated ATPase domains were used. For simulations, the homology model of the undocked conformation was based on the PDB-coordinates of the human Hsc70 (PDB: 3C7N), which contains the highest sequence homology to the nematode Hsc70. The docked conformation was based on the BiP structure (PDB: 5B84), which contains the highest sequence homology to the nematode Hsc70. Simulations were performed in a water box with the equivalent of 40 mM NaCl. After minimizing the water energy and temperature and pressure adaptation of the system, the molecular dynamics simulation was run to yield 1 ns trajectories according to the described procedure for Gromacs^36^. This calculation was performed on an Intel Core i7-6800K CPU @ 3.40 GHz with support of a NVIDIA GeForce GTX 1080 graphics chip using Gromacs 5.1.4 built in the 64 bit environment of Windows 10. This system was capable of producing 30 ns/day. Alternatively the CoolMUC linux cluster of the Leibnitz-Rechenzentrum (LRZ) Garching was used with Gromacs 2016. Calculations were performed systematically from customized *sbatch* scripts and usually employed 56 cores (2 nodes), capable of producing ~50 ns/day.

Initial trajectory analysis was performed with the Gromacs *energy* and *distance* tools. Simultaneous comparison of many 1 ns trajectories regarding distances between specific atoms for different Hsc70 variants in the two conformations were done by the customized script dynaMIX, which added the targeted atoms to each index.ndt file and initiated the distance determination for each time point with the Gromacs *distance* tool or with VMD^37^. Data points for distance histograms summarizing 30 1 ns time courses were collected from the last 20 frames of each trajectory. Resulting distance data were then plot in Origin 8.6. Images of specific amino acid positions were generated in VMD.

## Results

### Hsc70 S321F shows increased oligomerization and reduced nucleotide responses

Several mutations in Hsc70 were reported to suppress the *unc-23(e25)* and *unc-23(ok1408)* locomotion phenotype *in vivo*^17,20^. All these mutant sites (D233N, S321F, A379V and D384N) reside in the ATPase domain and the neighboring linker of Hsc70 (Fig. 1A). To obtain information on the mechanism of suppression, we purified these Hsc70 variants and performed biochemical characterization. Interestingly, all mutations support viability of *C. elegans*, which provides the powerful opportunity to biochemically characterize these non-lethal Hsc70 mutations and their molecular function.

Of the four mutants, only Hsc70 S321F showed an increased aggregation propensity during protein purification, while the other mutations behaved wild type like. To test, whether the mutations affect the stability of Hsc70 we performed thermal stability assays (Fig. 1B). Melting temperature for wild type Hsc70, Hsc70 D233N and D384N were observed at 39 °C, 39 °C and 38 °C respectively. Thus, thermal stability in these two mutants is not affected. Hsc70 A379V was slightly destabilized and already unfolded at 36 °C. Interestingly, the S321F mutation showed an increased melting temperature at 46 °C. Addition of nucleotides has been shown to exert a strong stabilizing effect on Hsc70 before^8^. Indeed, the melting temperature of Hsc70 increased to 51 °C after addition of 1 mM ADP. To test the response of the Hsc70 mutations to addition of nucleotides the thermal stability was assessed. Hsc70 D233N and D384N unfold at the same temperature as the wild type protein at 51 °C (Fig. 1C). Hsc70 A379V again is slightly destabilized unfolding at 49 °C. The ATP-induced stabilization suggests that the interaction with nucleotides is maintained in the three mutants (D233N, D384N and A379V) despite the mutations being in the nucleotide binding domain (see Fig. 1A). The only mutation that behaved differently was Hsc70 S321F. The addition of ADP did not result in any additional stabilization of the protein. As the melting temperature of Hsc70 S321F was higher than that of Hsc70 in the absence of nucleotides this protein appears more stable than Hsc70 in the absence and less stable in the presence of nucleotide (Fig. 1B,C).

We next tested the quaternary structure of the Hsc70 mutants. To this end we performed sedimentation velocity experiments with analytical ultracentrifugation (Fig. 1D). Here, wild type Hsc70 as well as Hsc70 D233N, A379V and D384N sediment with an identical sedimentation coefficient of 4.3 S ± 0.2 S. UltraScan-analysis suggests a molecular mass of roughly 70 kDa for the main species, showing that these purified proteins are mostly monomers in solution. A sedimentation coefficient of 4.3 S also matches values reported for the human Hsc70 protein in the past^38^. Hsc70 S321F, instead, sediments with 14 S, which reflects the formation of oligomers. The width of the distribution function in the dc/dt plots suggests the presence of diverse oligomeric forms. We performed SEC-HPLC to confirm this result. Indeed, for the variants with normal stability properties we observed a retention time of 19.5 minutes. Instead two distinct peaks with retention times of 14 and 15.5 minutes imply heterogeneous oligomers for Hsc70 S321F (Fig. 1E). In summary, the mutations D233N, A379V and D384N do not abolish nucleotide binding capacity and maintain oligomerization properties of wild type Hsc70. In contrast, Hsc70 S321F affects oligomerization, stability and possibly nucleotide binding suggesting large conformational rearrangements.

### ATP hydrolysis is inhibited in Hsc70 mutants

**Figure 2:**
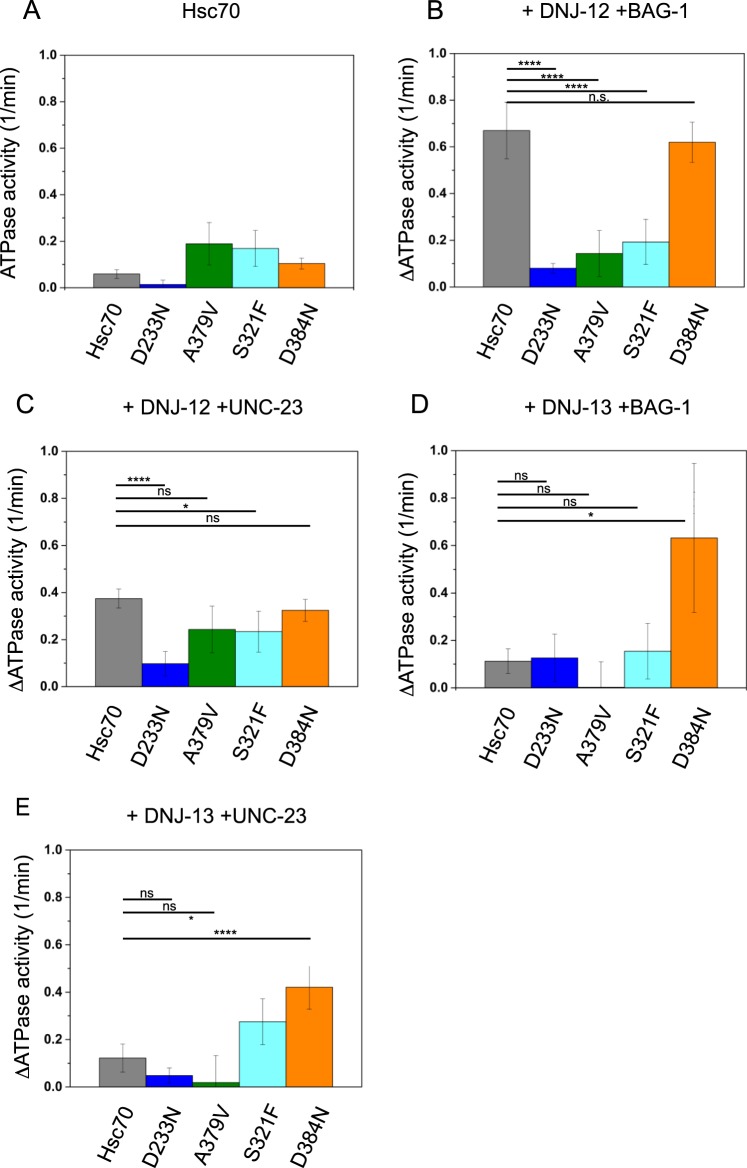
Stimulation of ATPase activity of Hsc70 variants. The ATPase activity of Hsc70 and mutant versions of it was determined in a regenerative ATPase assay as described in the Materials and Methods section. 3 µM Hsc70 were analyzed in standard buffer at 25 °C. (**A**) ATPase activity of the Hsc70 wildtype and mutants. (**B**) The Hsc70 ATPase stimulation after addition of DNJ-12 and BAG-1. The unstimulated ATPase activity was subtracted to provide a background-free value. 1 µM DNJ-12 and 3 µM BAG-1 was added to the Hsc70 mutants. (**C**) The Hsc70 ATPase stimulation after addition of 1 µM DNJ-12 and 3 µM △258 UNC-23. (**D**) The Hsc70 ATPase stimulation after addition of 1 µM DNJ-13 and 3 µM BAG-1. (**E**) The Hsc70 ATPase stimulation of 1 µM DNJ-13 and 3 µM △258 UNC-23. ****Indicates p < 0.001, *indicates p < 0.01, n.s. is not significant.

A central question is if essential functions of Hsc70 are affected by the mutations as all four mutations are able to suppress the strong locomotion phenotype induced by *unc-23* mutations *in vivo*^17,20^. The mutations are localized in the ATPase domain of Hsc70, thus we tested if ATP hydrolysis is affected leading to modified ATPase cycles. Wild type Hsc70 exhibits ATP turnover rates of 0.07 min^−1^. The mutation D233N showed significantly decreased ATP turnover to 0.02 min^−1^ (Fig. 2A), while S321F, A379V and D384N Hsc70 preparations showed slightly increased ATP hydrolysis.

In the presence of Hsp40 proteins and nucleotide exchange factors (NEFs) the ATP turnover of wild type Hsc70 can be strongly stimulated^8^. Individual cofactors stimulate the ATPase activity of the Hsc70 variants only mildly (Table 1). The strongest ability to stimulate the ATPase activity of Hsc70 is seen by combining the NEF BAG-1 and the Hsp40 like protein DNJ-12. Indeed, wild type Hsc70 can be stimulated approximately 10-fold, increasing its ATPase activity to 0.74 min^−1^ (ΔATPase = 0.67 min^−1^, Fig. 2B). While Hsc70 and Hsc70 D384N exhibited a similar stimulation, the ATPase activity of Hsc70 D233N and Hsc70 A379V was only mildly accelerated. In fact, the observed stimulation was only 0.08 min^−1^ and 0.12 min^−1^ respectively and thus much lower than that for wild type Hsc70 (Fig. 2B). Also, the S321F mutation was weakly stimulated upon addition of DNJ-12 and BAG-1, implying that under the conditions of the ATPase assay, this variant shows some functionality.

**Table 1:** ATPase turnover measured for mutated Hsc70 variants in combination with excess with 3 µM BAG-1, 1 µM DNJ-12, 1 µM DNJ-13 or 3 µM UNC-23.

	BAG-1	DNJ-12	DNJ-13	UNC-23
Hsc70	0.61 ± 0.05	0.29 ± 0.03	0.56 ± 0.06	0.18 ± 0.03
Hsc70 D233N	0.14 ± 0.06	0.33 ± 0.05	0.08 ± 0.04	0.12 ± 0.00
Hsc70 S321F	N.A.	N.A.	N.A.	N.A.
Hsc70 A379V	0.21 ± 0.15	0.07 ± 0.01	0.19 ± 0.13	0.02 ± 0.00
Hsc70 D384N	0.11 ± 0.03	0.34 ± 0.03	0.39 ± 0.18	0.24 ± 0.02

Activity values are derived from replicates and depicted values are given in min^−1^. Only the extent of stimulation is shown, as the unstimulated activity of the respective mutant and the background activity of the cofactor are subtracted as described in Materials and Methods section. Hsc70 S321F was omitted due to very high background activity.

An interesting question is how other cofactor combinations affect the ATPase activity. We next tested the influence of UNC-23 in combination with DNJ-12. We previously purified and characterized a stable fragment of UNC-23, termed Δ258-UNC-23^7^, which binds to Hsc70 and performs the nucleotide exchange reaction^7^. This fragment was now used in ATPase assays alongside DNJ-12. Hsc70 wild type was stimulated by 0.40 min^−1^ and all mutants were stimulated to a similar extend with the exception of Hsc70 D233N, which showed significantly reduced stimulation (Fig. 2C). To test the ability of different co-chaperones to stimulate the ATPase activity, the Hsc70 mutants were also subjected to the J-domain protein DNJ-13. ATPase cycle acceleration in combination with DNJ-13 yields in lower stimulation^7^. Interestingly, in combination with the NEF BAG-1, all mutants behave similar except for Hsc70 D384N. Here, the ATPase cycle is strongly stimulated in the presence of DNJ-13 and BAG-1 (ΔATPase = 0.64 min^−1^) while the other mutations, including Hsc70, Hsc70 D233N and Hsc70 S321F show only weak increases in ATP turnover (Fig. 2D). The strong stimulation of Hsc70 D384N is again observed with DNJ-13 and UNC-23 as NEF (Fig. 2E). These results show that all identified *unc-23* suppressor mutations in Hsc70 exhibit an altered functional interaction with Hsc70 co-chaperones despite being stable proteins with mostly normal nucleotide binding capabilities. This is observable as a mostly decreased stimulation for Hsc70 D233N and Hsc70 A379V and an increased stimulation of Hsc70 D384N. Thus, Hsc70 ATPase cycle regulation is differentially affected in the mutations while general ATP hydrolysis is possible in all mutants.

### NEF binding is conserved in Hsc70 mutants

**Figure 3:**
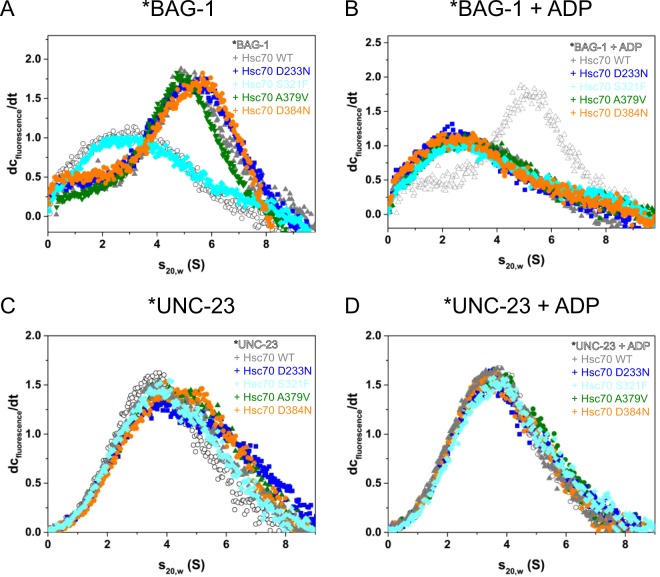
Influence of suppressor mutations on NEF interaction. Binding of Hsc70 mutants to co-chaperones via analytical ultracentrifugation. The conditions are listed in the Material and Method section. (**A**,**B**) Binding of *BAG-1 to wild type/mutant Hsc70 analyzed by analytical ultracentrifugation. *BAG-1 alone sediments at 2.2 S. Hsc70-bound *BAG-1 is observed at 4.9 S. (**A**) BAG-1 sedimentation either alone (open gray symbols) or in the presence of 2 µM Hsc70 (colored gray symbols), Hsc70 D233N (blue), Hsc70 S321F (cyan), Hsc70 A379V (olive) and Hsc70 D384N (orange). (**B**) Binding of *BAG-1 to wild type/mutant Hsc70 in the presence of ADP using the same color code as before. (**C**,**D**) Binding of *△258 UNC-23 to mutant/wild type Hsc70 analyzed by analytical ultracentrifugation. (**C**) Binding of 300 nM *△258 UNC-23 to wild type/mutant Hsc70 in the absence of nucleotides using the same color code as described above. (**D**) Binding of 300 nM *△258 UNC-23 to wild type/mutant Hsc70 in the presence of ADP using the same color code as above.

We next asked where the influences on the ATP turnover originate. To scrutinize, which of the co-chaperones is responsible for the observed changes, we tested their binding capability to Hsc70 individually. To this end we first employed analytical ultracentrifugation with fluorescently labeled BAG-1 (*BAG-1). *BAG-1 alone sediments at 2.2 S and in the presence of Hsc70 its sedimentation coefficient increased to 4.9 S. This indicates that *BAG-1 binds strongly to wild type Hsc70^8^. Likewise, all mutants bind *BAG-1 with the exception of Hsc70 S321F (Fig. 3A). This implies that the high affinity for BAG-1 is persevered in the three Hsc70 ATPase domain/linker mutations (D233N, D384N and A379V). To assess the full functionality of the *BAG-1/Hsc70-complex we tested if nucleotide addition reduces this protein-protein interaction. Indeed, after addition of ADP, the complex formation is diminished for all Hsc70 mutants similar to wild type Hsc70 (Fig. 3B). In conclusion, NEF interaction is functional and the response to nucleotide binding is mostly unaffected in the ATPase domain/linker mutations D233N, D384N and A379V (Fig. 3B).

In order to test for NEF specificity or if other NEFs behave similarly with respect to complex formation, we analyzed the binding of the Hsc70 mutants to the NEF *Δ258-UNC-23. To this end we employed analytical ultracentrifugation with fluorescently labeled UNC-23 (*Δ258-UNC-23). This fragment sediments with an s_20,w_ of 3.5 S. After addition of Hsc70, the resulting protein complex can be observed with an increased sedimentation coefficient (4.2 S) (Fig. 3C). Here, as reported before, binding is much weaker compared to BAG-1^7^, but detectable at Hsc70 concentrations of 3 µM. Hsc70 S321F again shows no binding to *Δ258-UNC-23, while the other mutants behave similar to wild type Hsc70 (Fig. 3C). All Hsc70 mutants similar to the Hsc70 wild type diminish their binding to *Δ258-UNC-23 in the presence of 2 mM ADP as to be expected for a nucleotide exchange factor interaction (Fig. 3D). Thus, while the ability of the Hsc70 mutants to increase their ATP turnover in the presence of co-chaperones is compromised, the binding of NEFs to the Hsc70 mutants is mostly conserved and still responsive to nucleotides in the mutants that are structurally least affected. This is particularly interesting, as NEFs as well as nucleotides bind the ATPase domain of Hsc70 and all mutations reside within this domain. Thus, the mutations do not induce large conformational rearrangements that abolish NEF binding.

### J-protein binding is strongly reduced in Hsc70 mutants

**Figure 4:**
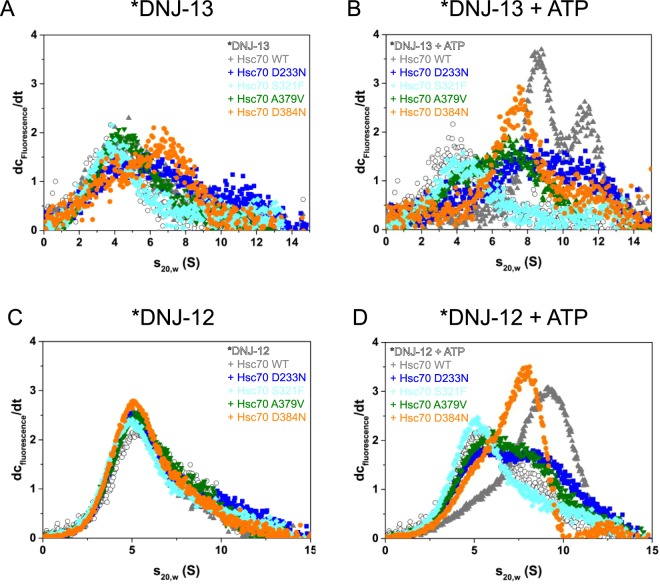
Influence of suppressor mutations on Hsp40-interaction. Binding of Hsc70 mutants to DNJ proteins via analytical ultracentrifugation. The conditions are as is listed in the Material and Method section. (**A,B**) Binding of *DNJ-13 to wild type/mutant Hsc70 analyzed by analytical ultracentrifugation. (**A**) Binding of 600 nM *DNJ-13 to wild type/mutant Hsc70 in the absence of nucleotides. DNJ-13 sedimentation was recorded either alone (open gray symbols) or in the presence of 4 µM Hsc70 wild type (gray), Hsc70 D233N (blue), Hsc70 S321F (cyan), Hsc70 A379V (olive) and Hsc70 D384N (orange). (**B**) Binding of *DNJ-13 in the presence of ATP to wild type/mutant Hsc70. Color code is as described above. (**C,D**) Binding of *DNJ-12 to wild type/mutant Hsc70 analyzed by analytical ultracentrifugation. (**C**) Binding of 300 nM *DNJ-12 to wild type/mutant Hsc70 in the absence of nucleotides using the same color code as described above. (**D**) Binding of 300 nM *DNJ-12 to wild type/mutant Hsc70 in the presence of ATP using the same color code as described above.

The drastic changes and the failure to stimulate mutant Hsc70’s ATPase activity by co-chaperones can barely be explained by the NEF interaction for three of the mutants as they behave very similar to wild type Hsc70. Thus, we next tested the binding of Hsp40-proteins to the Hsc70 mutants. We examined a labeled DNJ-13 (*DNJ-13) in analytical ultracentrifugation assays and tested its interaction with these Hsc70 mutants. We first addressed the formation of *DNJ-13/Hsc70 complexes in the absence of nucleotide for each Hsc70 mutant. Wild type Hsc70 does not form complexes with *DNJ-13 under these conditions (Fig. 4A). Similarly, no binding of Hsc70 S321F and A379V to *DNJ-13 was observed. Interestingly, some complex formation of Hsc70 D384N and very weak complex formation of Hsc70 D233N is detected (Fig. 4A). We previously reported very weak binding in the absence of ATP and strong formation of multimeric Hsc70/DNJ-13 complexes in the presence of ATP^8^. Indeed, in the presence of ATP, large *DNJ-13-containing complexes were formed with wild type Hsc70 (Fig. 4B). Hsc70 under these conditions showed distinct hetero oligomeric complexes at 8.4 S and 11.8 S. In turn, Hsc70 D384N formed one *DNJ-13-Hsc70 complex species sedimenting at 7.5 S. In both cases very little free *DNJ-13 at 4 S was observable (Fig. 4B). The interaction of *DNJ-13 with Hsc70 A379V and D233N was strongly reduced, resulting in a broad peak in the range of 6 to 8 S. In contrast to the strongly binding Hsc70 wild type and D384N, Hsc70 A379V retains a substantial amount of apparently free not complexed *DNJ-13. Interestingly, Hsc70 S321F showed no binding to *DNJ-13 in the presence of ATP (Fig. 4B). This demonstrates that DNJ-13 and the Hsc70 ATPase domain/linker mutants can form different oligomeric forms in the presence of ATP. However, the oligomer formation occurs most efficiently with wild type Hsc70. The interaction with DNJ-13 is reduced for all four Hsc70-variants. To test whether this effect is conserved in other Hsp40 proteins, we tested the binding to labeled DNJ-12 (*DNJ-12). Here no complex formation can be observed in the absence of nucleotide (Fig. 4C). In the presence of ATP instead, large protein complexes form at 9.9 S with Hsc70 wild type. Similarly, Hsc70 D384N is able to bind *DNJ-12 which results in complexes at 8.2 S. Again, the mutants A379V and D233N showed weak but reproducible binding to *DNJ-12, resulting in a shoulder of the dc/dt plot between 8 and 9 S. Nevertheless, in the presence of Hsc70 A379V and D233N most *DNJ-12 sediments with 4 S, representing unbound *DNJ-12 (Fig. 4D). Thus, complex formation between DNJ proteins and the Hsc70 ATPase domain mutations in the presence of ATP is decreased for all four mutations (Fig. 4D). Potentially, this highlights functional similarity between the different ATPase domain and linker mutations.

### Hsc70 mutants refold luciferase at lower yields

**Figure 5:**
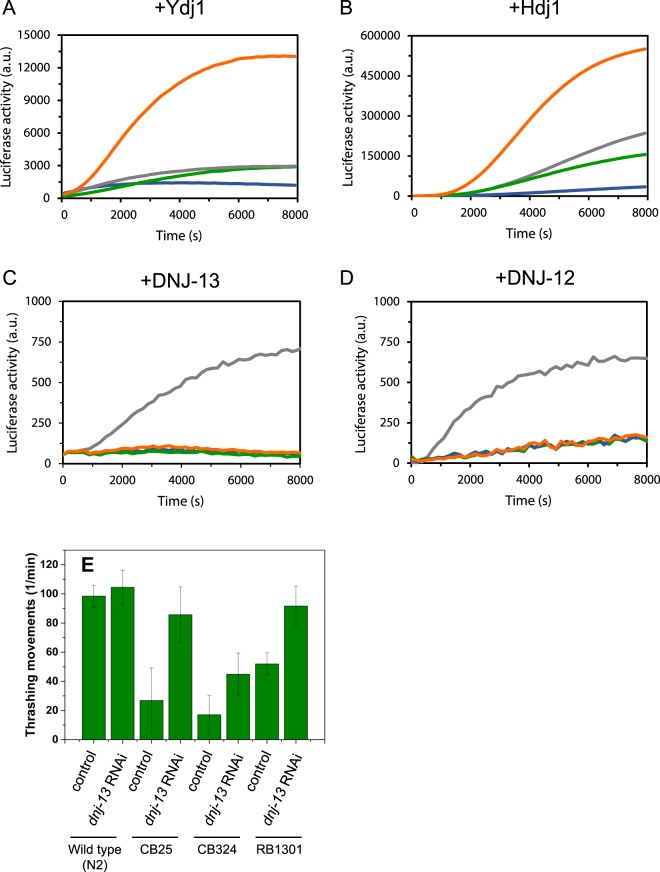
Influence of *Hsc70* ATPase domain mutation on refolding capacity. Refolding of denatured luciferase by mutant and wild type Hsc70s in the presence of the Hsp40-like cofactors Ydj1 (**A**), Hdj1 (**B**), DNJ-13 (**C**) as well as DNJ-12 (**D**). Luciferase is chemically denatured and refolded in refolding buffer as indicated in Material and Methods. Hsc70 wild type (gray), Hsc70 D233N (blue), Hsc70 A379V (olive) and Hsc70 D384N (orange) are shown. (**E**) *dnj-13* RNAi rescues the motility defect in three different *unc-23* mutations (CB25 *unc-23(e25*), CB324 *unc-23(e324)*, RB1301 *unc-23(ok1408)*). Thrashing movements per minute of *unc-23* mutated nematodes in M9 buffer. Nematodes were grown on control and *dnj-13* RNAi conditions from hatching on and assessed at adult day 1. n = 20.

We finally tested, whether the strong differences in cofactor binding are reflected in the capability of the Hsc70 mutants to actively refold substrates. This activity requires the close cooperation between Hsp40-proteins, substrate and Hsc70 *in vitro*, but no correlation exists between ATP turnover and efficiency of the folding process. Based on the observed viability of the nematodes harboring these mutations *in vivo*, it is to be expected that the essential functions of Hsc70 can be fulfilled despite the mutations. We performed luciferase refolding assays with Hsc70 and the yeast Hsp40-protein Ydj1, which is able to cooperate with higher eukaryotic Hsc70s^39,40^. Wild type Hsc70 efficiently refolds luciferase (Fig. 5A). Interestingly, Hsc70 D384N refolds even higher amounts of luciferase than the wild type protein. However, Hsc70 A379V and more strikingly Hsc70 D233N show diminished refolding capability (Fig. 5A). We then tested, whether the human Hsp40 protein Hdj1 and the nematode DNAJA-homolog DNJ-12 and the nematode DNAJB-homolog DNJ-13 also show differences in stimulating the luciferase refolding reaction. Together with Hdj1, Hsc70 D384N shows the highest refolding activity and Hsc70 D233N again the weakest. For DNJ-12 and DNJ-13, which are much less active this trend seems to not be conserved, as here the wild type Hsc70 shows the highest activity, indicating that the efficiency of the folding reaction is a product of each component and their interactions. In conclusion, the interaction with J-domain proteins is sufficient to allow some Hsc70 folding activity in these mutants. Given that *C. elegans* can live based on these mutant Hsc70-proteins, the residual folding activity apparently is sufficient to enable folding of Hsc70’s essential clients *in vivo*.

### Depletion of dnj-13 suppresses the phenotype of unc-23(e25)

We have observed reduced DNJ-13 binding for most of the Hsc70 mutants in our study. An interesting question is whether the reduction of DNJ-13 and Hsc70 also rescues the strong locomotion phenotype in *C. elegans*. For the RB1301 strain we had previously observed that a reduction of cellular DNJ-13 levels is conferring resistance against the locomotion defect caused by the mutant allele *unc-23(ok1408)*, while reduction in *dnj-12* and *dnj-19* did not cause a change^7^. The Hsc70 S321F and Hsc70 D384N mutants were identified as suppressors of this allele. Despite the difficulty to compare *in vitro* and *in vivo* data, it is interesting to note that our *in vitro* data find that both mutants show decreased DNJ-13 affinity. In addition, especially Hsc70 D233N and Hsc70 A379V, which were identified to rescue the locomotion phenotype of *unc-23(e25)*, show diminished DNJ-13 binding. We thus tested, whether the *unc-23(e25)* allele’s phenotype, which is based on a lysine to glutamate exchange in position 297 of UNC-23 in the strain CB25, can be suppressed by reducing cellular DNJ-13 levels *in vivo*. To this end we performed RNAi experiments with the strain CB25 depleting *dnj-13* under the same conditions used to suppress the phenotype of the RB1301 strain. Nematodes harboring the *e25-*allele show progressively reduced motility at adult day 1. Applying *dnj-13* RNAi improved the locomotion significantly (Fig. 5B). RNAi against the homolog genes *dnj-12* and *dnj-19* on the other hand did not have an effect on the phenotype of CB25 nematodes (data not shown). We also tested the strain CB324, harboring the allele *unc-23(e324)*. These nematodes showed a stronger phenotype compared to the other strains, but they likewise showed an improvement in motility, if DNJ-13 is depleted. This demonstrates that the genetic interaction between *unc-23* and *dnj-13* previously observed in *unc-23(ok1408)* nematodes^7^ is preserved in other *unc-23* mutated strains. Thus, the positive impact of lower DNJ-13 levels is observable in several alleles of *unc-23* and potentially explains the selection of Hsc70 variants with diminished DNJ-13-binding as suppressors of this phenotype.

### unc-23 suppressor mutations in Hsc70 affect the communication within the ATPase domain

**Figure 6:**
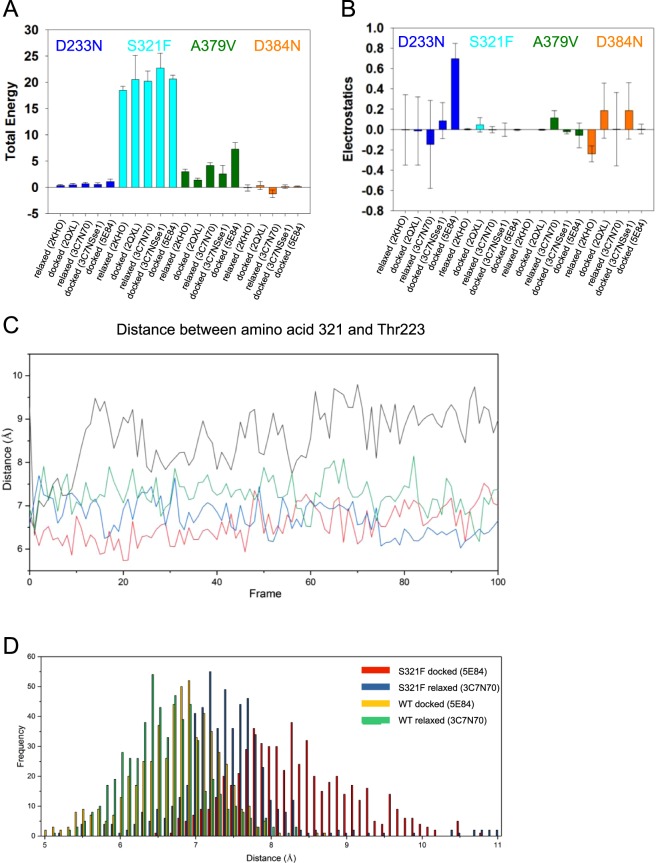
Simulated impact of the Hsc70 mutations on structure and stability. (**A**) Influence of the mutations on the stability of Hsc70 based on the FoldX BuildModel tool. An ensemble of 5 structures was generated by Chimera for each of the starting PDB structures. In each of these structures the amino acid exchanges were introduced and the FoldX tool returned estimated penalty values for the introduction of the mutation. The standard deviation results from differences within these 5 ensemble structures. (**B**) Influence of the mutation type on the charged interactions/electrostatics as indicated by FoldX. (**C**) Excerpt from molecular dynamics trajectories for Hsc70 S321F, representing the distance between the Cα of Thr223 and amino acid 321. Wild type Hsc70 open: green, wild type Hsc70 closed: red, Hsc70 S321F open: blue, Hsc70 S321F closed: black. (**D**) Average distances within the last 20 frames based on 30 simulations each. The plots show wild type Hsc70 and Hsc70 S321F (both either based on the ATP-complexed BIP or the uncomplexed Hsc70 structure).

Of the four Hsc70-variants the S321F-mutation appears most compromised in its biochemical function, while the other mutations do not affect stability and nucleotide binding to a great extent. Nevertheless, they also show striking changes to their interaction with DNJ-13. To estimate the impact of the mutations on protein structure and stability, we used molecular modeling. We determined structures for nematode Hsc70 in the two conformations of Hsc70: undocked/nucleotide-free/substrate-lid-closed and docked/ATP-bound/substrate-lid-open. Several publicly available structures for each conformation can be used for this. The docked Hsc70s were based on the crystal structures of human BIP in complex with ATP (PDB: 5E84, see Fig. 1A right) and on yeast Sse1 either alone (PDB: 2QXL) or in complex with bovine Hsc70 (PDB: 3C7N). In all these structures the Hsc70-like protein was crystallized in a conformation, where the substrate lid is docked onto the ATP-binding site and the SBD is accessible. The relaxed structures are based on bovine Hsc70 (PDB: 3C7N) and on *E. coli* DnaK (PDB: 2KHO, see Fig. 1A left), where the two domains of Hsc70 are separated and the SBD is closed by the substrate-lid. The similarities between the generated docked structures for nematode wild type Hsc70 are high (RMSD of 0.05 to 0.16 nm) and the modeled conformations are almost superimposable in the ATPase domain. This is similar for the undocked structures (RMSD of 0.09 to 0.3 nm). (Fig. 6).

We used the isolated ATPase domains to test for energetic influences that may originate from the four mutations. Based on the FoldX BuildModel tool, which we used to generate and characterize the mutations, the S321F ATPase domain is destabilized by roughly 20 kJ/mole in all five PDB-based model structures (Fig. 6A). In Hsc70 S321F the exchange from Ser321 to Phe321 may be so severe that several aspects of Hsc70 are affected including structure and stability. The fact that the nematodes can live based on the S321F mutation demonstrates a surprising plasticity apparently allowing *C. elegans* to complement such strong impairments to its sole and essential Hsc70 homolog.

The A379V-mutation is also destabilized but to a much lesser extent, while the other two mutations are almost neutral in all structures tested. Nevertheless, D233N in the ATP-induced conformation based on PDB: 5E84 shows a loss of electrostatic energy contribution, which implies that charged interactions at the mutation site could be affected (Fig. 6B). This is not observable to the same extent in the D384N mutation, which affects the same surrounding amino acids, but does so in a surface-exposed linker region. Thus, especially Hsc70 S321F and A379V are destabilized compared to the wild type protein, while D233N shows altered electrostatic interaction.

To get more information on structural influences of mutations that reside within the ATPase domain, we tested short molecular dynamics (MD) simulations. To this end we used the ATPase domain of the closed structure based on the human Hsc70 (PDB: 3C7N) and the ATPase domain of the docked structure based on BIP (PDB: 5E84). With each of the mutations we simulated short trajectories, which can provide insight into the local flexibility and structural deformation caused by the amino acid exchanges. We then analyzed the development of selected interatomic distances in the recorded trajectories. Indeed, we can see that based on the Cα-atoms of S321 (atom 4954 in Hsc70) and the nearby Cα of Thr223 (atom 3398 in Hsc70) a weak deformation of the beta sheet can be induced (Fig. 6C). We performed 30 such simulations with the first homologs of the modeled structures and compared the distance distributions. Indeed, a prominent difference can be observed in the histograms, as the S321F version generates higher distances within its structures during the simulation (Fig. 6D). The bulky Phe-residue introduced at position 321 differs drastically from the smaller Ser 321, which could account for these higher distances.

**Figure 7:**
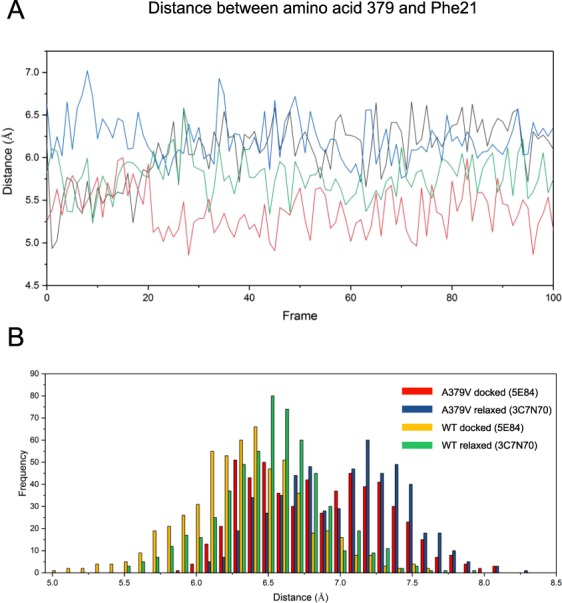
Simulated impact of the A379V mutation. (**A**) Excerpt from molecular dynamics trajectories for Hsc70 A379V, representing the distance between the Cα of amino acid 379 and Phe21. Wild type Hsc70 open: green, wild type Hsc70 closed: red, Hsc70 A397V open: blue, Hsc70 A397V closed: black. (**B**) Average distances between the Cα atoms of amino acid 379 and Phe21 within the last 20 frames in Hsc70 or Hsc70 A379V either based on the ATP-complexed BIP or Hsc70 alone.

Likewise, the change of Ala to Val at position 379 induces local distortions that lead to a slightly increased distance between the Cα of Ala379 (atom 5841 of Hsc70) and the Cα of Phe21 (atom 286 in Hsc70), which is nearby (Fig. 7A). This is more obvious in histograms summarizing 30 trajectories (Fig. 7B). Thus, these mutations may induce local distortions of the packed structure surrounding the mutation site, which then could affect the protein’s functionality as observed in our biochemical characterization.

**Figure 8:**
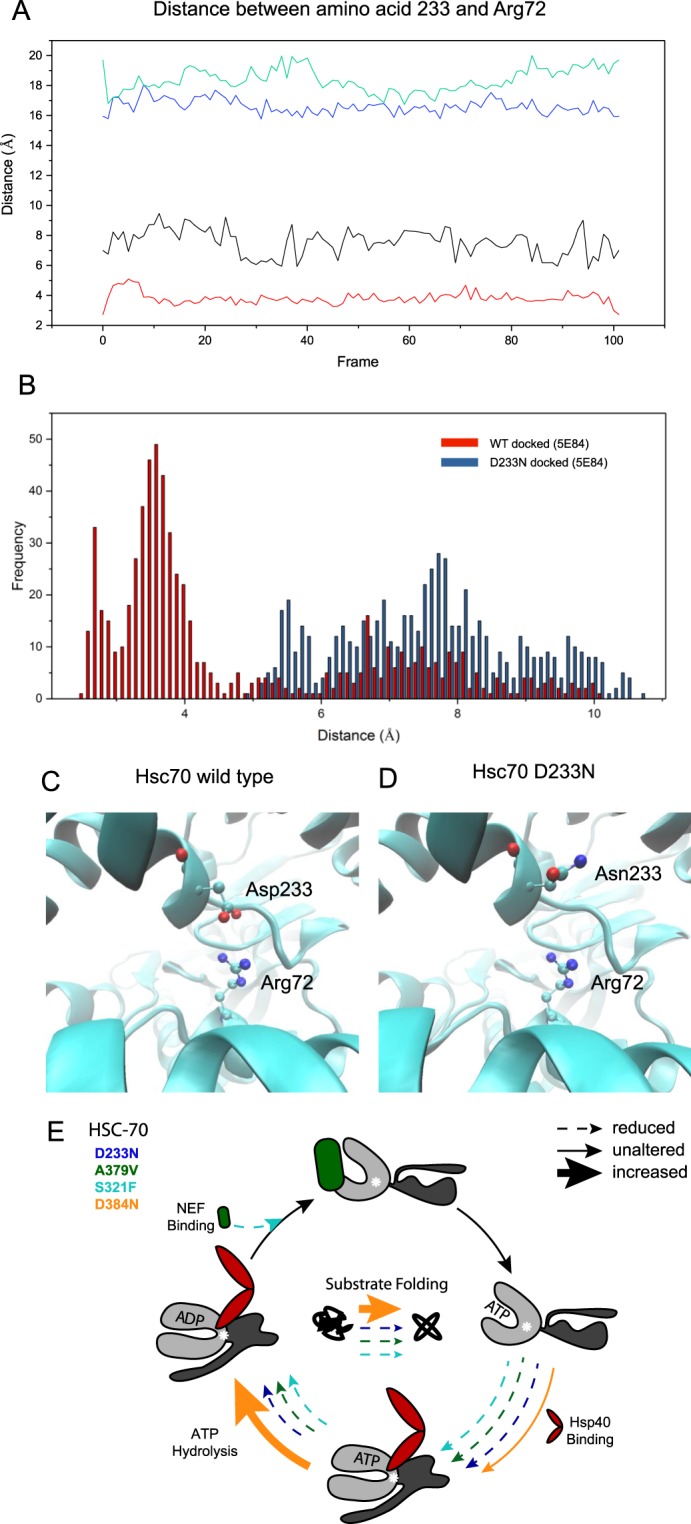
Simulated impact of the Hsc70 D233N mutation on the distance to Arg72. (**A**) Excerpt of the trajectories for Hsc70 D233N representing the distance between the Cγ of Glu/Gln233 and the Cε of Arg72. Wild type Hsc70 open: green, wild type Hsc70 closed: red, Hsc70 D233N open: blue, Hsc70 D233N closed: black. (**B**) Distances between the last 20 frames between Asp233 (Hsc70) or Asn233 (Hsc70 D233N) and Arg72 based on the ATP-complexed BIP structure. (**C**) Positioning of the salt bridge between Asp233 and Arg72 (CeHsc70 based on the structure PDB:5E84). (**D**) Positioning of the amino acids Asn233 and Arg72 in the D233N mutation of Hsc70. (**E**) Summary of the Hsc70 cycle. The colors reflect the respective mutants: Hsc70 D233N (blue), Hsc70 S321F (cyan), Hsc70 A379V (green) and Hsc70 D384N (orange). Solid arrow: not altered, dashed arrow: reduced, bold arrow: increased. Steps that are not analyzed are not represented by arrows. Summary is shown for the ATPase assay with DNJ-12 and Bag-1 as cofactors, which yields in the highest stimulation in wild type. Summary is shown for the luciferase refolding assay with Ydj-1 and Hdj-1, which yields in the highest activity in wild type.

In contrast, the changes caused by D233N appear more defined. No obvious structural effects are observable and there is no size difference between the original and replacing amino acid. Measuring the distance between the Cγ of D233 (atom 3525 in Hsc70) and the Cε of Arg72 (atom 1074 in Hsc70), it becomes obvious that these two side chains can form a salt bridge in the closed structure (Fig. 8A). The distance here fluctuates between 3 and 4 Å. In the open, nucleotide free structure the distance between these two side chain atoms is fluctuating between 16 and 20 Å, as they are residing now in opposite sides of the opened nucleotide binding region. In the closed state Asp233 is located in one lobe of the ATP-binding domain, while the acceptor of the negative charge, Arg72, resides in the other lobe and they approach each other once these lobes get closer after ATP-binding. It is interesting to note that Asp233 is conserved in Hsc70 proteins and also Arg72 is strictly conserved in all Hsc70 proteins tested here. It is obvious that this mutation site is far away from the proposed binding site of Hsp40’s J-domain, thus most likely an indirect effect caused by altered conformational changes explains the weakened Hsp40 affinity observed *in vitro*. Even though the binding of the nucleotide appears to be uncompromised, the subsequent conformational changes might be affected due to the removal of the salt bridge. When the D233N variant is used in MD-simulations, the distance between Asn233 and Arg72 is increased to roughly 6–10 Å in many closed structures (Fig. 8B,C), implying that the Asn side chain is not able to contribute a similarly productive interaction. In the wild type Hsc70 the salt bridge instead is fairly stable (Fig. 8B,D). Given that Hsc70 D233N fails to generate the ATP-induced, J-domain binding conformation in our biochemical experiments, this mutation apparently leads to an Hsc70 variant, which has an altered ATPase cycle resulting from impeded conformational changes. These results provide rationality on how the mutation at the distant position 233 can alter the binding of the J-domain protein by reducing the closing efficiency of the ATPase lobe and thereby reducing the overall ATPase rate and the affinity of the DNJ-cofactor requiring this conformation. This makes the Hsc70 D233N protein a very interesting mutant to further investigate the conformational mechanism of Hsc70 in a variant, which still is functional *in vivo* and supportive to life.

## Discussion

In this study we characterize the biochemical properties of four Hsc70 mutations within the ATPase domain and neighboring linker mutations, which can act as suppressors of the *head-bent* phenotype in *C. elegans in vivo*. These four mutations show different biochemical properties regarding stability, cochaperone binding and activity (Fig. 8E) and the structural changes in these variants are sufficiently strong to induce energetic penalties in structural simulations. Based on our biochemical analysis, all these mutations cause changes to Hsc70’s interaction with the J-domain proteins DNJ-13 and DNJ-12, in most cases by reducing the affinity (D233N, S321F and A379V, Fig. 8E). This might be caused by steric clashes in the ATPase domain in Hsc70 S321F and A379V. The D233N mutation possibly inhibits nucleotide-induced conformational changes which are required for Hsp40 binding. Thus, in Hsc70 D233N, S321F and A379V Hsp40 binding and subsequently the functional activities are inhibited.

This is not observed for the D384N mutant, where an increased ATPase stimulation is present. Interestingly, ATPase stimulation does not strictly correlate with refolding activity in this mutant. It is apparently possible that D384N becomes inactive when the ATPase activity is higher than usual. The mode of action to rescue the head-bent phenotype may be directly related to the changes of the analyzed Hsc70 cycle or may involve further interacting proteins. Previous studies have shown that cooperativity between nematode Hsp40 proteins increases refolding *in vitro* and decreases protein aggregation *in vivo*^9,41^. Thus, one exciting possibility is that cooperativity between Hsp40 proteins is altered in the Hsc70 mutants. Future studies which characterize the molecular mechanism of cooperation will shed light on this possibility.

Conditions which reduce the interaction with the DNJ-13 cofactor are helpful to overcome the severe phenotypes associated with *unc-23* mutations *in vivo*. This may be achieved by depleting *dnj-13* by RNA interference^7^, or in cases where DNJ-13 is present this may also be achievable by modifications to Hsc70. The depletion of cellular *dnj-13* has been successful to suppress the *unc-23* deletion mutant allele *ok1408*^7^. Here we report that *dnj-13* depletion likewise rescues the single point-mutant allele *e25*, which harbors the mutated UNC-23 variant UNC-23 E297K and in addition the *unc-23(e324)* allele. Of note, while DNJ-12 and DNJ-13 interaction are reduced *in vitro* only *dnj-13* depletion *in vivo* rescues the muscular attachment phenotype. It is possible that DNJ-13 binds a specific client subset that directs Hsc70 away from the muscular attachment sites and that DNJ-12 would lack this feature.

Nevertheless, all Hsc70 mutants are still functional *in vivo*. The changes observed *in vitro* suggest that protein interactions are likewise going to be reshaped *in vivo*, but to an extent, which does not impede livability. The observed changes to the DNJ-13 interaction might be enough to generate a nematode strain, which is resistant to the *unc-23* mutation E297K and the debilitating phenotype associated with it. Thus, a diminished J-domain interaction of Hsc70 may reduce the detrimental muscular attachment phenotype. In this respect it is also interesting to note, that in humans the Hsp40 protein DNAJB6 and the nucleotide exchange factor Bag3 are relevant in hereditary forms of muscular dystrophy^42–46^. Even though it is unclear, whether similar cellular mechanisms are responsible, this shows that in mammalian cells the Hsc70 system is participating in the development and suppression of related pathologies.

## Data Availability

All data are fully available without restriction.
